# Dietary Starch Concentration Affects Dairy Sheep and Goat Performances Differently during Mid-Lactation

**DOI:** 10.3390/ani11051222

**Published:** 2021-04-23

**Authors:** Mondina Francesca Lunesu, Mauro Decandia, Giovanni Molle, Alberto Stanislao Atzori, Giovanni Cristoforo Bomboi, Antonello Cannas

**Affiliations:** 1 Dipartimento di Agraria, Sezione di Scienze Zootecniche, Università degli studi di Sassari, Viale Italia 39, 07100 Sassari, Italy; asatzori@uniss.it (A.S.A.); cannas@uniss.it (A.C.); 2 Agris Sardegna, Loc. Bonassai, 07100 Sassari, Italy; mdecandia@agrisricerca.it (M.D.); gmolle@agrisricerca.it (G.M.); 3 Dipartimento di Medicina Veterinaria, Sezione di Biochimica, Università degli studi di Sassari, Via Vienna 2, 07100 Sassari, Italy; gcbomboi@uniss.it

**Keywords:** non-fiber carbohydrates, milk production, body reserves, ewes, goats, insulin, growth hormone, fat, ruminants

## Abstract

**Simple Summary:**

In order to favor milk production, this research suggests that the concentration of starch in the diet of dairy ewes in mid-lactation should be reduced, compared to early lactation, by introducing sources of highly digestible fiber. On the other hand, this type of diet is not adequate for dairy goats, which should maintain the use diets rich in starch, even in mid-lactation, to support milk yield, as well.

**Abstract:**

Evolution of milk production, body reserves and blood metabolites and their relationships with dietary carbohydrates were compared in 30 Sarda dairy ewes and 26 Saanen dairy goats in mid-lactation. From 92 to 152 ± 11 days in milk (DIM), each species was allocated to two dietary treatments: high-starch (HS: 20.0% starch, on DM basis) and low-starch (LS: 7.8% starch, on DM basis) diets. In mid-lactating goats, the HS diet increased fat-corrected milk yield (FCM (3.5%); 2.65 vs. 2.53 kg/d; *p* = 0.019) and daily milk net energy (NE_L_; *p* = 0.025), compared to the LS diet. The body condition score (BCS) was not affected. In mid-lactating ewes, the LS diet increased FCM (6.5%) (1.47 vs. 1.36 kg/d; *p* = 0.008), and NE_L_ (*p* = 0.008), compared to the HS diet. In addition, BCS was greater in HS than in LS ewes (3.53 vs. 3.38; *p* = 0.008). Goats had a higher growth hormone (GH) and lower insulin concentration than ewes (GH: 2.62 vs. 1.37 ng/mL; *p* = 0.04; insulin: 0.14 vs. 0.38 µg/L; *p* < 0.001 in goats and ewes, respectively). In conclusion, in mid-lactation, the two species responded differently to dietary carbohydrates, probably due to differences in the concentration of GH and insulin. The HS diet favored milk yield in goats and body reserve accumulation in ewes. In ewes, the partial replacement of starch with highly digestible fiber increased energy partitioning in favor of milk production.

## 1. Introduction

Among small ruminants, the effect of high non-fiber carbohydrates (NFC, i.e., an estimate of the sum of simple sugars, starch and pectins) diets on milk production during early lactation, when the energy balance is often negative, is well documented. In dairy ewes, diets rich in starch (>20–30%, on DM basis) almost always reduce the energy deficit and increase milk production, as reviewed by Cannas et al. [1]. Only when very high-quality forages, such as those with highly degradable fiber and low lignification produced in most northern or southern countries, are used does the role of NFC become less relevant [2]. Similarly to sheep, in highly productive dairy goats, which normally use 60–85% of total glucose for milk synthesis, most of the time at onset lactation, only high-starch diets can guarantee adequate glucose availability and energy supply [3]. Much less is known about mid- and late lactation, when the energy balance of the goats and ewes is usually positive.

In dairy ewes, some experiments showed that diets rich in highly digestible fiber (e.g., high content of beet pulps or soybean hulls) increased milk production, whereas high-starch diets stimulated fattening [4,5,6]. Conversely, a positive effect of highly digestible fiber in mid- and late lactation has not been observed in goats, which also responded positively to high-starch diets in this lactation stage [7,8]. This is in full contrast to what was observed in dairy ewes in the same lactation stage. These different responses could be due to species differences, innate or owing to genetic selection; in the hematic concentration of hormones (e.g., growth hormone (GH) and insulin); or in the responsiveness of the tissues involved in energy partitioning and blood glucose utilization in mid- and late lactation. Other explanations could be differences in acetate and glucose requirements for milk synthesis because of the different fat to lactose ratio in the milk of the two species [1] or the better ability of goats to divide starch-rich diets into small and more frequent meals compared to ewes [9], which could therefore diminish insulin spikes. However, these hypotheses are merely speculative, because, to our knowledge, no studies have compared the responses of ewes and goats to different levels of starch and digestible fiber fed in the same experimental conditions and lactation stage.

Thus, the objectives of this study were to test if dietary NFC content, especially starch, can impact milk production and body reserve accumulation in mid-lactation, when goats and ewes could be more prone to insulin action, and if ewes and goats are affected differently by dietary starch and, consequently, fiber concentration.

## 2. Materials and Methods

The experiment was conducted at the Bonassai experimental farm, located in the northwest of Sardinia (40° N, 32° E, 32 m a.s.l), of the Agricultural Research Agency of Sardinia (AGRIS, Sardinia), Italy.

The animal protocol described below was done in compliance with the EU and Italian regulation on animal welfare, and all measurements were taken by personnel previously trained and authorized by the institutional authorities managing ethical issues at the University of Sassari. Experimental procedures with animals (sheep and goats) were approved by the Animal Care and Use Committee of the University of Sassari and Agris, Italy (CIBASA 10.12.2014)

### 2.1. Experimental Procedure: Animals and Diets

A 90-day preliminary period (from parturition to 91 ± 11 days in milk (**DIM**)) was conducted before the administration of the experimental diets. During this period, all animals were fed the same high-starch diet used later in the experimental period. More details concerning the preliminary period were previously described by Lunesu et al. [10]. At the end of the preliminary period, and hence from 92 ± 11 DIM, 30 mature Sarda dairy ewes and 26 mature Saanen dairy goats were allocated to two dietary groups: high-starch (**HS**; 15 ewes + 13 goats) and low-starch (**LS**; 15 ewes + 13 goats) groups. Subgroups were balanced within species to have the same average body condition score (**BCS**; goats: 2.84 vs. 2.79; ewes: 3.39 vs. 3.33 in HS vs. LS diet, respectively) and milk production (goats: 3.13 vs. 3.12 kg/d; ewes: 1.76 vs. 1.78 kg/d in HS vs. LS diet, respectively).

The experiment lasted 60 days (from 92 to 152 ± 11 DIM). From 92 to 139 DIM, the animals were kept in a closed barn in 4 large pens (2 pens/species, 68.4 m^2^/pen). Each pen had access to an external paddock (54 m^2^/paddock). Each pen had a water trough with fresh and clean water, with adequate drinking space for all animals. At the end of this period, for 12 days (from 140 to 152 ± 11 DIM) animals were kept in metabolic cages.

All animals were fed a diet (Table 1) containing 29% of chopped dehydrated alfalfa, 4% of mature ryegrass hay and 67% of experimental pellets (as-fed basis).

The experimental pellets differed as follows: (i) for the HS group, a high-starch pellet with 28.1% starch and 30.7% NDF, and (ii) for the LS group, a low-starch pellet with 10.0% of starch and 48.8% of NDF (on DM basis). The pellets differed, mainly, because most of the corn meal and all the barley meal of the high-starch pellet were replaced with soybean hulls, a high source of highly digestible fiber, in the low-starch pellet (Table 1). Sodium bicarbonate and magnesium oxide were included in both diets, even though probably not necessary in the LS diet, to avoid any possible bias due to the buffers. In addition, all animals were fed whole corn grain during the two daily milking sessions (100 g/d as fed, in total).

The two diets were iso-nitrogenous, whereas the carbohydrate concentration (NFC and NDF) differed between the two groups, as showed in Table 1.

The diets were group fed ad libitum and supplied twice a day immediately after each milking (0700 h; 1500 h). Pellets and chopped dehydrated alfalfa were mixed together and supplied in a large manger, to which all animals had free access, whereas mature ryegrass hay was supplied in another manger at the same time. Orts were quantified daily to guarantee at least 10% excess in the diet supplied, compared to the actual intake every day.

At the end of the experiment, during the last 12 days (from 140 to 152 ± 11 DIM), in vivo digestibility trial was carried out in order to measure individual feed intake and digestibility of the diets. Twenty ewes and 20 goats (for each species, 10 from the HS diet and 10 from the LS diet) were kept in metabolic cages for 12 days (7 days of adaptation period to the cage and 5 days of measurements). The animals were randomly selected to be representative of their species dietary groups in terms of milk yield, BCS and DIM.

### 2.2. Measurements and Sampling

During the period spent in the barn pens, orts derived from pellets, chopped dehydrated alfalfa mix and mature ryegrass hay were collected and weighted separately every day. Samples of feeds and orts were collected weekly for subsequent chemical analysis.

During the digestibility trial, the orts were weighted daily and individually throughout the adaptation and measurements periods to estimate voluntary feed intake. Daily orts of each animal were collected, pooled and then subsampled at the end of the trial for subsequent analysis. After the adaptation period, during the 5 sampling days of the experimental period, feces were collected each day at 0800 h, weighted and then mixed, and an aliquot (10% of their total fresh weight) was sampled and immediately stored at −20 °C until chemical analysis.

Milk production was measured individually once a week, and individual milk samples (12 samples/animal; in total, 672 milk samples) were collected and immediately stored at 4 °C until analysis could be carried out (within 2 days from the sampling).

Individual BCS and body weight (BW) were assessed every two weeks. BCS was estimated by 3 trained investigators who assessed the degree of fattening around the lumbar region using a 0- to 5-point scale, with minimum intervals of a quarter of a point, according to the Russel et al. [11] method. BW was measured by using an electronic scale before the morning meal.

At 126 and at 134 ± 11 DIM, after milking but before the morning meal, blood samples were collected from the jugular vein in vacuum tubes with anticoagulants and immediately centrifuged at 3500 rpm for 10 min at 4 °C to separate plasma, which was collected and stored at −20 °C until the samples were assayed.

### 2.3. Chemical Analyses

#### 2.3.1. Feedstuffs, Orts and Feces

The samples of feeds, orts and feces were ground with a Hammer mill by using a 1 mm screen and then analyzed for DM, CP (Kjeldahl), NDF, ADF, ADL (including termostable α-amylase), ether extract (Soxlet), starch (polarimetric method [12]) and ash after drying at 105 °C. Before grinding, the fecal samples were thawed and oven-dried at 60 °C for 48 h. The dietary ingredients and the orts rich in starch were pretreated overnight with 8 m urea. The NDF and ADF values were quantified on an ash-free basis. The NFC was calculated with the following formula: NFC = 100–CP–NDF–ash–ether extract.

#### 2.3.2. Milk

The morning and afternoon milk samples were analyzed separately for fat, protein (N × 6.38), lactose (infrared method; Milkoscan 4000, Foss Eletric, Hillerød, Denmark), urea content (enzymatic-colorimetric method based on Berthelot reaction; Chemspec 150, Bentley Instruments Inc., Chaska, MN, USA) and somatic cell count (SCC, flow-cytometry method; Fossomatic 5000, Foss Electric, Hillerød, Denmark). Fat-corrected milk yield (FCM) was calculated separately for the two species. For ewes, milk production was normalized at 6.5% fat as FCM (6.5%) = 0.37 × milk yield (kg/d) + 9.7 × milk fat (%) × milk yield (kg/d), according to the equation developed by Pulina et al. [13]. For goats, milk production was normalized at 3.5% fat as FCM (3.5%) = 0.63 × milk yield (kg/d) + 10.5 × milk fat (%) × milk yield (kg/d), according to Pulina et al. [14]. Daily milk net energy (NEL, Mcal of NE/d) was calculated as NEL = (251.73 + 89.64 × PQ + 37.85 × (PP/0.95)) × Yn/1000, for ewes, and NEL = (289.72 + 71.93 × PQ + 48.28 × (PP/0.92)) × Yn/1000 for goats, according to Tedeschi et al. [15]. In particular, Yn is measured milk yield at a particular day of lactation (kg/d), PQ is measured milk fat at a particular day of lactation (%), PP is measured true milk protein for a particular day of lactation (%).

#### 2.3.3. Blood Samples

Blood samples were analyzed for glucose, non-esterified fatty acids (NEFA), urea, GH, insulin, leptin and insulin growth factor-I (IGF-I).

Glucose, NEFA and urea were analyzed by enzymatic colorimetric assays in both species. Insulin and leptin were analyzed through a solid phase two-site enzyme immunoassay based on the direct sandwich technique by using ELISA kit (Mercodia AB, Uppsala, Sweeden for insulin and Blugene Biotech, Shanghai, China for leptin).

Plasma concentrations of GH and IGF-I were evaluated by radio-immuno assay (RIA) technique.

### 2.4. Statistical Analysis

Data on milk production and composition, BW and BCS were analyzed within animal species by the PROC MIXED procedure of SAS (Version 9.0, SAS Institute Inc., Cary, NC, USA) with repeated measurements. A mixed model was used to test the differences between the diets to analyze data concerning the mid-lactation period, as reported as follows:

*Y_ijk_* = *μ* + *α_i_* + *β_j_* + *αβ_ij_* + *γ* + *π_ij_* + *ε_ijk_*

where *Y_ijk_* is the dependent variable, *μ* is the general mean, *α_i_* is the effect of diet (*i* = HS, LS), *β_j_* is the effect of period (*j* = from 92 to 139 DIM), *αβ_ij_* is the diet × period interaction (*i* = HS, LS; *j* = from 92 to 139 DIM), *γ* is the random effect of animal, *π_ij_* is the covariate and *ε_ijk_* is the residual error. Data concerning the last day of the preliminary period were included in the model as covariate. Somatic cell count was log transformed.

Data on digestibility trials (from 140 to 152 DIM) were analyzed by the PROC GLM procedure of SAS (Version 9.0, SAS Institute Inc., Cary, NC, USA) to test the differences between species and diets and their interactions.

Data on plasma metabolites and hormones were analyzed by the PROC MIXED procedure of SAS (Version 9.0, SAS Institute Inc., Cary, NC, USA) with repeated measurements to test the effect of diet (HS, LS), period, species (ewes, goats) and their interactions.

All data were expressed as mean ± SEM. Means were separated using Tukey’s test. The accepted level of significance was *p* < 0.05.

## 3. Results

### 3.1. Feed Composition and Intake

As planned, the HS diet had a higher NFC and starch concentration than the LS diet (Table 1). The LS diet had a higher NDF, ADF, ADL and ash concentration, compared to the HS diet (Table 1). The diet intake, accounting for orts quantity and composition, differed little, compared to the diet supplied in both species. Indeed, dietary CP concentration of the diet actually eaten was slightly lower (goats: HS = 15.2%, LS = 15.0%; ewes: HS = 15.2%, LS = 15.4%) than that of the diets supplied (HS = 15.5%, LS = 15.6%; Table 1). Dietary NDF concentration was similar (goats: HS = 36.8%, LS = 50.6%; ewes: HS = 37.0%, LS = 50.2%), compared to that of the diets supplied (HS = 36.7%, LS = 48.8%; Table 1). In contrast, starch dietary concentration was slightly higher (goats: HS = 24.2%, LS = 10.5%; ewes: HS = 24.1%, LS = 10.5%), compared to that of the diets supplied (HS = 20.0%, LS = 7.8%; Table 1).

**Figure 1:**
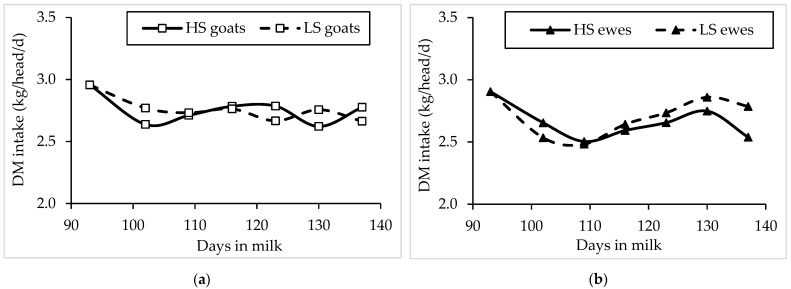
Evolution of dry matter (DM) intake (kg/d) in mid-lactating Saanen goats (**a**) and Sarda ewes (**b**) fed high-starch (HS) and low-starch (LS) diets. The symbols indicate the mean of the daily measurements of the group fed DM intake.

Group average DM intake was fairly constant in goats during the experiment, while it decreased, then increased, and then decreased again in sheep over time. On average, the two groups of goats had very similar DM intake (goats: HS and LS= 2.75 kg/d; average values of the data reported in Figure 1a), whereas the average group DM intake in the ewes was numerically lower for the HS than for the LS diet (HS = 2.66 kg/d, LS = 2.71 kg/d; average values of the data reported in Figure 1b). The intake of DM and nutrients was also similar between species.

In contrast to the group average values, individual DM intake measured during the digestibility trial was significantly and markedly higher (*p* < 0.001) in goats, compared to ewes, while it was not affected by dietary treatment (Table 2). The in vivo digestibility coefficients did not differ between species (Table 2). The HS diet had the highest DM apparent digestibility (*p <* 0.001), whereas the LS diet had a higher NDF true digestibility than the HS diet (*p <* 0.001) (Table 2).

### 3.2. Milk Production and Composition

#### 3.2.1. Effect of Diet

**Figure 2:**
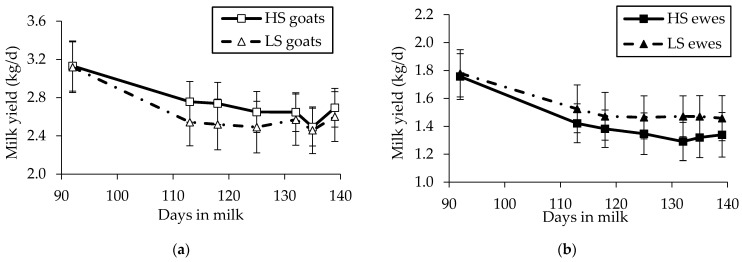
Evolution of milk yield (kg/d) in mid-lactating Saanen goats (**a**) and Sarda ewes (**b**) fed high-starch (HS) and low-starch (LS) diets. The vertical lines at each symbol indicate the SEM of the values.

In Saanen goats, milk yield decreased in both dietary groups during the trial (Figure 2a) and was higher in the HS than in the LS group (2.66 vs. 2.53 kg/d ± 0.04 (mean + SEM); *p* = 0.011; Table 3). The FCM (3.5%) and NE_L_ were also higher in the HS than in the LS group (FCM (3.5%): 2.65 vs. 2.53 kg/d ± 0.05, *p* = 0.019; NE_L_: 1.88 vs. 1.80 Mcal NE/d ± 0.03, *p* = 0.025). Milk fat concentration was lower in HS-fed goats than in LS-fed goats (3.50 vs. 3.64%; ± 0.06; *p* = 0.031), whereas milk fat yield did not differ between the two diets. Milk protein concentration did not differ between HS and LS diets, whereas milk protein yield was greater in goats fed the HS diet than goats fed the LS diet (*p* = 0.033).

In Sarda ewes, milk yield decreased in both groups during the trial (Figure 2b) and was not statistically different between the two diets but was numerically greater in the LS than in the HS group (1.44 vs. 1.38 kg/d ± 0.04; Table 4). Fat-corrected milk yield and NE_L_ were higher in the LS than in the HS group (FCM (6.5%): 1.47 vs. 1.36 kg/d ± 0.04; *p =* 0.008; NE_L_: 1.53 vs. 1.41 Mcal NE/d ± 0.04; *p =* 0.008). The ewes fed the LS diet had a higher milk fat concentration (6.68 vs. 6.41% ± 0.07; *p =* 0.001), milk fat yield (*p =* 0.002), milk protein concentration (5.16 vs. 5.06% ± 0.04; *p* = 0.014) and milk protein yield (*p =* 0.018), compared to the ewes fed the HS diet.

#### 3.2.2. Effect of Period, Diet × Period Interaction, and Covariate

In goats, the effect of period was statistically significant for NE_L_, milk fat concentration, milk fat yield, milk protein concentration, milk lactose concentration, SCC and milk urea concentration (Table 3).

In ewes, the effect of period was statistically significant for milk fat (*p* = 0.047), milk protein and milk urea concentration (Table 4).

In goats, the diet × period interaction was not significant for any of the milk parameters considered (Table 3), whereas in ewes, the diet × period interaction influenced significantly milk urea (Table 4).

In both species, the covariate was statistically significant for all parameters considered (Table 3 and Table 4).

### 3.3. Evolution of Body Weight and Body Reserves

#### 3.3.1. Effect of Diet

**Figure 3:**
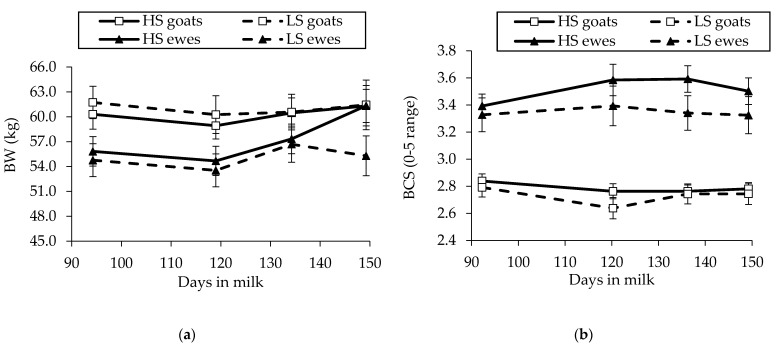
Evolution of (**a**) body weight (BW; kg) and (**b**) body condition score (BCS; 0–5 range) in mid-lactating Saanen goats and Sarda ewes fed high-starch (HS) and low-starch (LS) diets. The vertical lines at each symbol indicate the SEM of the values.

In Saanen goats, BW was not affected by dietary treatment (60.52 vs. 60.25 kg ± 0.45 for HS and LS, respectively). In HS-fed goats, BW slightly decreased from the first measurement (60.31 kg) but tended to increase afterwards, being slightly higher at the last measurement (61.31 kg). The same pattern was observed for BW in the LS goats (Figure 3a). In goats, BCS was not affected by dietary treatment (2.75 vs. 2.73 ± 0.03 for HS and LS, respectively) (Figure 3b).

In Sarda ewes, BW did not differ between diets (55.94 vs. 55.92 kg ± 0.45 for HS and LS, respectively) and increased over time in HS ewes (from 55.83 to 61.33 kg), while in LS ewes, it decreased from the first measurement, increased at 134 DIM, and then decreased till the end of the experiment (Figure 3a). By contrast, the BCS was greater in HS-fed ewes than in LS-fed ewes (3.53 vs. 3.38 ± 0.05; *p* = 0.008; Figure 3b).

#### 3.3.2. Effect of Period, Diet × Period Interaction, and of the Covariate

The effect of period was significant for BW (*p* = 0.03 and <0.001 for goats and ewes, respectively) but not for BCS. The diet × period interaction was not significant for BW and BCS in goats and ewes. The covariate was significant (*p* < 0.001) for both BW and BCS in goats and ewes.

### 3.4. Effects on the Metabolic and Hormonal Status during Mid-Lactation

The values related to the metabolic and hormonal status are reported in Table 5. Plasma glucose concentration was higher in ewes than goats (56.0 vs. 48.3 mg/dL ± 1.77 (mean + SEM); *p* < 0.001). Plasma NEFA concentration was greater in goats than ewes (0.17 vs. 0.12 mmol/L ± 0.02; *p* = 0.039). Plasma urea and IGF-I concentrations were not affected by species, but IGF-I was numerically greater in ewes than goats. Goats had higher plasma GH (2.62 vs. 1.37 ng/mL ± 0.58; *p* = 0.038) and leptin (24.7 vs. 12.0 ng/mL ± 2.18; *p* < 0.001) concentrations and lower insulin concentrations (0.14 vs. 0.38 μg/L ± 0.05; *p* < 0.001) than ewes.

In both species, the metabolic and hormonal status were not affected by the diets.

**Figure 4:**
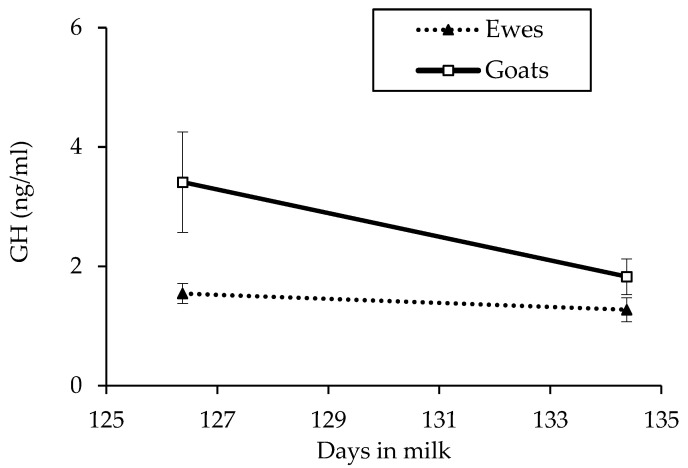
Graphical representation of species × period interaction (*p* = 0.04) concerning the growth hormone (GH) concentration detected in plasma of mid-lactating Saanen goats and Sarda ewes. The vertical lines at each symbol indicate the SEM of the values.

The effect of period and the species × period interaction (Figure 4) was statistically significant only for GH (*p* = 0.01 and 0.04; respectively).

In both species, the effect of the diet × period interaction was not significant for any of the plasma parameters considered. In addition, the diet × species interaction was significant (*p* < 0.016) only for NEFA.

## 4. Discussion

### 4.1. Intake and Digestibility

Group average DM intake was similar between dietary treatment for goats but not for ewes. The intake pattern in ewes clearly showed that, as the trial progressed, the DM intake of LS ewes increased progressively.

The evolution of DM intake observed in LS ewes is in agreement with the results of Zenou and Miron [17] and Cannas et al. [6]. The increase of DM intake in LS ewes could be due to the fact that the small NDF particle size of the pellets did not impose physical restrictions on the intake of the diet with a higher NDF level, thus allowing the ewes to exert a metabolic control of intake [6]. More specifically, DM intake was probably driven by milk production, and the LS ewes, having higher NE_L_ and FCM than the HS ewes, had a higher DM intake to compensate for their lower dietary energy concentration. This did not occur in goats, since milk production for the LS group was lower than for the HS group.

During the digestibility trial, carried out after the end of the production trial, DM intake was higher in goats than in ewes. During this trial, however, the environmental temperature increased dramatically, with the maximum temperature varying from 24 to 30 °C and the relative humidity reaching 100% for at least 50% of the time. This negatively affected milk production and dietary DM intake in both species, but especially in the ewes, which showed marked signs of discomfort and difficulties adapting to the metabolic cages. In general, ewes are less resistant to heat stress than goats [18]. In addition, the fact that ewes were much fatter (mean BCS: 3.46) than the goats (mean BCS: 2.75) certainly increased their heat stress.

In the digestibility trial, the lack of effect of diet on milk production and dietary DM intake is in contrast with the results of the production trial. An explanation could be that the high NDF concentration of the LS diets may have exacerbated the negative effects of the heat stress on milk production and DM intake, compared to the HS groups, because fiber fermentation and metabolic utilization produces more heat than starch [19,20], thus aggravating the heat stress of the animals. This effect seemed to be less strong in goats, probably due to their already-mentioned higher resistance to heat stress, compared to sheep. It is also possible that the difference in the two species in the partition of dietary energy between milk production and body reserves might have induced lower heat production and higher tolerance in goats.

**Figure 5:**
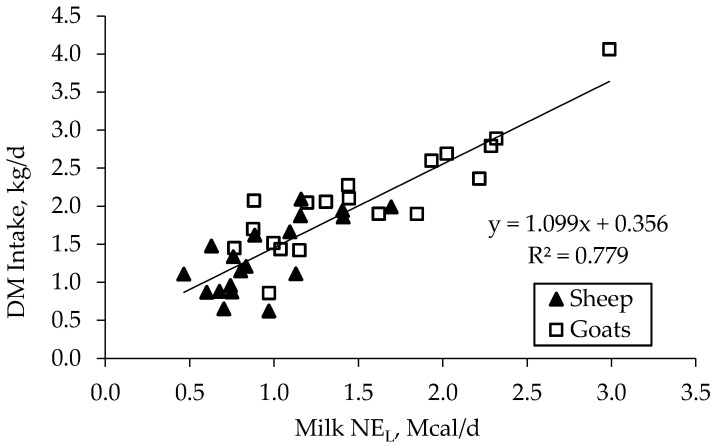
Relationship between individual daily DM intake and daily milk NE_L_ of the Saanen goats and Sarda ewes during the digestibility trial carried out in mid-lactation.

To better understand the relationship between DM intake and animals’ performances, the individual mean DM intake values measured during the digestibility trial were regressed against milk production (expressed as daily milk NE_L_ output to make the species comparison feasible; two outliers were discarded because of the extremely low milk production), milk composition and body reserves, including the experimental factors animal species and diet. The highest association was found between DM intake and milk NE_L_ (Figure 5):

DM intake (kg/d) = 0.356 + 1.099 daily milk NE_L_ (Mcal/d)

R^2^ = 0.779 SD residuals = 0.341 *n* = 38

and between net energy of lactation (NEL) intake and daily milk NE_L_:

NEL intake (Mcal/d) = 0.564 + 1.553 daily milk NE_L_ (Mcal/d)

R^2^ = 0.774 SD residuals = 0.49, *n* = 38.

The experimental factors animal species and diet were not significant, nor were BW and BCS. These results confirm the DM intake differences observed in the production trial reflected the differences in milk production (assessed as milk energy to make it more comparable among the treatments of the animals).

As expected, the HS diet was the most digestible, in accordance with previous studies [8,21]. The high NDF digestibility of LS diets observed in our experiment was detected in previous studies [6,17,21] and is certainly due to their high content of soyhulls, which have a highly digestible fiber.

### 4.2. Milk Production

The fact that the HS diet had a positive effect on daily milk production, FCM (3.5%) and NE_L_ in goats but a negative effect on FCM (6.5%), daily milk NE_L_ and, numerically, on milk yield in ewes is evidence that the two species under the study responded differently to the dietary treatments applied during mid-lactation.

The positive effect of the HS diet on mid-lactating Saanen goats found in our trial is in accordance with previous studies comparing high- and low-NDF diets in mid-lactating Sarda goats [7] and high- and low-starch diets (high-starch: starch 21.9%, NFC 28.7%, NDF 40.6%; low-starch: starch 7.0%, NFC 28.7%, NDF 46.5%, on DM basis) in mid-lactating Murciana–Granadina goats [8]. However, other authors observed no differences [21]. The positive effect (in terms of FCM and NE_L_) of the LS diet on mid-lactating ewes observed in our experiment is in accordance with previous studies conducted with ewes fed low-NFC–high-digestible fiber diets compared to ewes fed high-NFC diets [4,6,17]. Other studies reported a negative effect or no effect of high NFC-diets on milk production in ewes in mid-lactation [22].

### 4.3. Milk Composition

#### 4.3.1. Milk Fat

In our experiment, the HS diet reduced milk fat concentration in both species, despite the observed opposite effects of dietary treatments on milk yield between species. Some studies observed that diets rich in starch decreased milk fat, also, in other species, such as dairy cows [23], whereas diets rich in fiber increased milk fat. In fact, in dairy sheep, there is a positive relationship between NDF and milk fat concentration [24], and, when dietary starch was substituted by digestible fiber sources during the mid-late lactation in dairy ewes, a higher milk production and milk fat concentration occurred with high-NDF–high-digestible fiber treatments [6,17].

In Sarda goats in mid-lactation, milk fat concentration did not differ between low-NDF (NDF 36.9%, NFC 36.0%, on DM) and high-NDF (NDF 44.7 %, NFC 29.3%, on DM) diets [7]. In Murciana–Granadina goats (106 DIM), low-starch diets, increased milk fat concentration, compared to high-starch diets (low-starch diet: starch 7.0%, NDF 46.5%; high-starch diet: starch 21.9%, NDF 40.6%, on DM; 6.4 vs. 5.4% ± 0.17, respectively; *p* = 0.01) [8], even though this could partially be an effect of the decreased milk production observed with the low-starch diets.

#### 4.3.2. Milk Protein

In our experiment, the response in terms of milk protein differed between the two species. In goats, the HS diet did not affect milk protein concentration but increased milk protein yield, compared to the LS diet, whereas in ewes, milk protein concentration and yield were higher in the LS group. In a previous study on Sarda goats, milk protein concentration was higher with a low-starch diet than with a high-starch diet, probably due to the concentration effect associated with the lower milk production of low-starch diets [7]. In Murciana–Granadina goats, milk protein concentration did not vary between a high-starch and a low-starch diet [8,21]. Our results on ewes are in contrast with other studies [6]. Overall, it appears that milk protein concentration does not vary much between high- and low-starch diets when milk production is similar, whereas it increases in the treatments that reduce milk yield.

### 4.4. Evolution of Body Weight and Body Reserves

In goats, BW and BCS were unaffected by dietary treatment, in accordance with Cannas et al. [7]. In addition, as the experiment progressed, BW and BCS varied very little. In Sarda ewes, BW did not differ between the two diets but increased over time in both groups. By contrast, BCS was greater in HS-fed ewes than in LS-fed ewes and increased markedly over time in the HS group, while it did not change in the LS group.

Overall, our results suggest that, in lactating goats, at equal DM intake, the difference in energy intake between the HS diet, which had the highest energy concentration, and the LS diet induced higher milk production, with no effects on body reserve accumulation. In contrast, in lactating ewes, the HS diet partitioned more energy to body reserve accumulation than to milk production, whereas the LS diets, very rich in digestible fiber, seemed to have stimulated milk production and did not induce body reserve accumulation.

It is well known that BW is affected by the gut content, so it is a less reliable indicator of body reserve variations than BCS, particularly in adult animals. Thus, the subsequent discussion will focus on BCS variations.

As suggested by Boerman et al. [25], nutrient partitioning is driven toward fat deposition with high-starch diets (containing 33% corn grain, 32% starch, 3.2% fatty acids, 25% NDF) and toward milk production with low-starch-plus-fat diets (containing 2.5% palmitic acid-enriched fatty acid, 16% starch, 5.4% fatty acids, 33% NDF) in dairy cows. The same authors suggested that the use of low-starch-plus-fat diets in dairy cows could be the solution to reduce overweight during mid-lactation and, therefore, maintain milk yield. In general, in mid-late lactation, a high-starch diet has a positive and stronger effect on body weight, which tends to increase, than on milk production, which instead tends to decrease in dairy ewes [26].

The increase in BCS observed in ewes could be the cause of the heat stress that ewes suffered during digestibility trial in mid-lactation. In fact, it is possible that heat stress was exacerbated by the high BCS of the ewes and, to a lesser extent, of the goats.

### 4.5. Effect of the Utilization of High-Starch and Low-Starch Diet in Mid-Lactation on the Evolution of Metabolites and Hormones

The type of the diet (HS vs. LS) did not affect the metabolites and hormones studied in sheep and in goats. The absence of differences on glucose concentration between diets was not expected, since the HS diet should have promoted higher gluconeogenesis than the LS diet. Indeed, previous studies observed higher glucose concentration in mid-lactating Sarda sheep fed a high-starch diet, compared to a low-starch diet [5,6], although in both experiments, milk production was higher in the low-starch than in the high-starch diet. By contrast, in Saanen goats fed a no-forage diet low in NFC or a control diet high in NFC (28.8 and 40.6% NFC, respectively; on a DM basis), there were no differences in glucose concentration in mid- and late lactation [27].

Considering that the mammary gland is as glucose-dependent organ [28] and that glucose is the most important substrate used for lactose synthesis, the lower blood glucose concentration observed in goats was probably associated with a high utilization of glucose by the mammary gland, due to their much higher milk production and lactose yield, compared to ewes.

In both species, NEFA concentration, a good indicator of energy balance, did not differ between high- and low-starch diets, in accordance with Cannas et al. [6], in ewes, which had similar daily energy intake when fed high- and low-starch diets. Differently, Cannas et al. [5] found higher NEFA values in mid-lactating ewes fed a low-starch diet, compared to a high-starch diet, but, in this case, the low-starch diet induced lower daily energy intake and, thus, a less positive energy balance. In our study, NEFA concentration was lower in sheep, confirming the tendency of this species toward body fat deposition, as observed in the BCS evolution. The higher values of NEFA observed in goats confirm that this species had high energy drain to sustain milk production.

The lack of difference in blood urea between the HS and LS diets in both species was in line with what found for milk urea concentration.

The growth hormone concentration did not differ between the two diets in both species. In previous studies on sheep, GH was higher with a low-starch diet than a high-starch diet (1.43 vs. 1.23 ng/mL; *p* < 0.02) [5], probably because the energy intake and balance were either higher in the high-starch diet or did not vary between high-starch and low-starch diets (3.28 vs. 3.83 ng/mL, respectively; *p* > 0.2) [6], and the dietary energy intake was similar between treatments. Growth hormone concentration in mid-lactating goats was higher for high-amylose starch (the least degradable starch at rumen level) than for normal starch diets (2.55 vs. 1.65 ng/mL; *p* = 0.06) at equal dietary starch concentrations [29], suggesting that GH is increased by diets that provide more rumen escape starch and, thus, higher intestinal glucose absorption. The higher GH concentrations observed in goats than in ewes are probably associated with the higher milk yield and lower energy balance, as suggested by the lower BCS accumulation, of goats in our experiment, confirming the important galactopoietic activity of this hormone.

The lack of difference in insulin concentration between the two diets in both species is in contrast to previous studies, where high-starch diets increased insulin concentration, compared to low-starch diets in Saanen goats [30] and in Sarda ewes [5]. The higher insulin concentration observed in sheep than goats suggests that the former had a metabolic status more inclined toward an anabolic pathway than the latter and confirmed the inverse relationship between insulin and milk production level [31].

Insulin-like growth factor I did not differ between the two diets and between ewes and goats. In this regard, the literature is contrasting. Magistrelli et al. [30] observed a higher IGF-I concentration in Saanen goats fed a high-starch compared to low-starch diet, whereas another study [5] observed a higher IGF-I concentration in Sarda ewes fed a highly digestible fiber.

Leptin was not affected by the two diets in both species, as previously observed by Cannas et al. [5] in mid-lactating sheep and by Wang et al. [29] in dairy goats fed a normal or high-amylose starch at equal dietary starch concentrations. The lack of effect of diet could be related to the fact that leptin depends more on the overall energy balance than on the dietary source, as already observed by Cannas et al. [5]. Differences in leptin concentration observed between ewes and goats could be due to differences in body fat deposition (different sites used to depot body fat) between the two species. Scientific studies evidenced that different breeds selected for milk or meat production have a different body fat distribution, with a higher subcutaneous fat deposition in meat than in milk breeds, which, instead, have a higher visceral fat deposition [32,33,34]. Moreover, these differences occur not only among different breeds but also between goats and sheep [35]. For example, in our previous study [36], in which Sarda sheep and Saanen goats were studied simultaneously, we evidenced that, in goats, most of the body fat was deposed in the sternal region. In contrast, in ewes, body fat deposition occurred mostly around the tail and the lumbar regions. The differences in the fat deposition existing between the two species are probably the reason why some studies observed a different leptin expression in subcutaneous fat [37] than the abdominal fat [38] or visceral fat [39]. In addition, leptin is a hormone that contrasts lipogenesis, thus regulating the satiety sense [40]. In our opinion, sheep of our experiment accumulated much more body reserves than goats, probably because of their lower leptin concentration, suggesting a lower ability to limit energy intake when energy balance is positive.

The highest GH, NEFA and leptin concentration observed in goats confirmed the better aptitude of Saanen goats for milk production. By contrast, the higher glucose and insulin concentration observed in Sarda ewes confirmed the tendency of this species to depot body reserves.

## 5. Conclusions

In conclusion, ewes and goats in mid-lactation appear to respond differently to different types of carbohydrates (i.e., starch vs. digestible fiber) and to have a different nutrient partitioning toward milk or body reserves, probably due to their different metabolic and hormonal status. In particular, when considering milk production in mid-lactation, dairy goats take advantage of high-starch diets, whereas ewes benefit from low-starch and highly digestible fiber diets. The species differences observed might be peculiar of the breeds studied, and, more specifically, to their genetic merit. Therefore, other comparative studies considering different breeds and populations of different genetic merit are needed to better understand the mechanism behind the partitioning of nutrients in small ruminant species.

## Figures and Tables

**Table 1:** Ingredients and chemical composition of the high-starch and low-starch diets supplied during the experiment.

	HS Diet	LS Diet
Diet ingredients (% as fed) ^1^		
Pellet	67.0	67.0
Dehydrated chopped alfalfa	29.0	29.0
Mature ryegrass hay	4.0	4.0
TOTAL	100.0	100.0
Pellet ingredients (% as fed)		
Dehydrated alfalfa	30.0	30.0
Corn meal	21.1	3.0
Barley meal	13.4	0.0
Wheat bran	10.1	5.0
Soybean hulls	9.0	43.2
Soybean meal 44	5.0	7.4
Sugarcane molasses	4.6	4.6
Sodium bicarbonate	3.0	3.0
Bentonite	2.0	2.0
Magnesium oxide	1.5	1.5
Minerals and vitamins	0.3	0.3
Appetizer (commercial mix)	0.03	0.03
Chemical composition ^2^		
DM (% as fed)	89.6	89.1
CP (% DM)	15.5	15.6
Ash (% DM)	11.0	11.2
Ether extract (% DM)	1.4	1.4
NDF (% DM)	36.7	48.8
ADF (% DM)	25.6	35.5
ADL (% DM)	4.7	5.1
NFC (% DM) ^3^	35.4	23.0
Starch (% DM)	20.0	7.8

HS = High-starch diet; LS = Low-starch diet, DM = Dry matter. ^1^ Additional supply of whole corn grain: 100 g/d in mid-lactation with the following chemical composition: DM = 86.5%, as fed; on a DM basis: CP = 8.0%, ash = 1.43%, NDF = 16.7%, NFC = 71.8%, starch = 69.6%. ^2^ The chemical composition does not include the corn grains supplied at milking. ^3^ NFC: 100–CP–ash–NDF-ether extract.

**Table 2:** Intake, level of intake, in vivo digestibility coefficients and milk yield response to high-starch and low-starch diets fed to mid-lactating ewes and goats during the digestibility trial (from 140 to 152 ± 11 DIM; *n* = 38; two outliers, one sheep and one goat, were discarded because of the extremely low milk production).

					Effect		
Item	Diet	Goats	Sheep	SEM	Species	Diet	SxD ^2^
DM intake (kg/d)	HS	2.3	1.4	0.2	***	ns	ns
	LS	2.0	1.2				
DM intake (% of BW)	HS	3.7	2.7	0.3	**	0.08	ns
	LS	3.1	2.1				
DM apparent digestibility (%)	HS	68.5	68.0	0.7	ns	***	ns
	LS	64.3	64.2				
NDF true digestibility (%)	HS	50.2	53.2	1.0	0.1	***	ns
	LS	58.3	58.8				
ME concentration (Mcal/kg of DM) ^1^	HS	2.3	2.3	0.02	ns	***	ns
	LS	2.2	2.2				
Milk yield (kg/d)	HS	2.47	0.96	0.2	***	ns	ns
	LS	2.13	0.85				

HS = High-starch diet; LS = Low-starch diet; BW = Body weight; ME = Metabolizable energy. ** indicate *p* < 0.01, *** *p* < 0.001, ns *p* > 0.10. ^1^ Calculated as ME = 0.82 × digestible energy (DE), according to Cannas et al. [16]. ^2^ Species × diet interaction.

**Table 3:** Evolution of milk yield, fat-corrected milk yield, net energy of lactation, fat, protein, lactose, somatic cell count and urea in mid-lactating Saanen goats fed high-starch and low-starch diets.

		Last Day of the Preliminary Period	Experimental Period (from 92 to 139 ± 11 DIM)								Effect			
	Diet		1	2	3	4	5	6	Mean ^3^	SEM ^4^	Diet	Period	D × P ^1^	π ^2^
Milk yield (kg/d)	HS	3.13	2.75	2.73	2.64	2.64	2.49	2.69	2.66	0.04	*	ns	ns	***
	LS	3.12	2.55	2.52	2.49	2.57	2.46	2.60	2.53					
FCM (3.5%) (kg/d) ^5^	HS	3.15	2.80	2.69	2.65	2.60	2.49	2.69	2.65	0.05	*	0.06	ns	***
	LS	3.23	2.67	2.53	2.50	2.48	2.46	2.56	2.53					
NE_L_ (Mcal NE/d) ^6^	HS	2.24	1.99	1.90	1.87	1.84	1.76	1.91	1.88	0.03	*	*	ns	***
	LS	2.30	1.89	1.79	1.77	1.76	1.74	1.81	1.80					
Fat (%)	HS	3.6	3.7	3.4	3.5	3.4	3.5	3.5	3.5	0.06	*	***	ns	***
	LS	3.9	4.1	3.7	3.7	3.3	3.7	3.5	3.6					
Fat (g/d)	HS	112.5	101.4	91.3	93.0	89.0	87.7	94.5	92.8	2.1	ns	***	ns	***
	LS	121.0	102.0	89.7	88.9	82.3	87.0	87.6	89.6					
Protein (%)	HS	3.2	3.2	3.1	3.1	3.1	3.1	3.2	3.1	0.02	0.09	*	ns	***
	LS	3.2	3.2	3.2	3.2	3.2	3.1	3.2	3.2					
Protein (g/d)	HS	99.4	88.1	85.0	82.6	81.3	77.1	84.9	83.2	1.5	*	0.06	ns	***
	LS	99.7	81.2	79.4	78.7	81.2	76.8	81.7	79.8					
Lactose (%)	HS	4.5	4.5	4.4	4.4	4.4	4.4	4.4	4.4	0.01	*	*	ns	***
	LS	4.6	4.4	4.4	4.3	4.4	4.4	4.4	4.4					
SCC (log)	HS	3.0	3.2	2.8	3.0	3.0	3.0	2.9	3.0	0.05	ns	**	ns	***
	LS	2.9	3.1	2.9	3.1	3.0	2.9	2.8	3.0					
Urea (mg/dL)	HS	39.9	48.3	45.7	46.7	39.9	44.1	39.1	44.0	0.8	*	***	ns	***
	LS	43.5	41.9	42.8	45.0	39.4	42.5	38.5	41.7					

HS = High-starch diet; LS = Low-starch diet; DIM = Days in milk; FCM = Fat-corrected milk yield; NE_L_ = Daily milk net energy; NE = Net energy; SCC = Somatic cell count. * indicates *p* < 0.05, ** *p* < 0.01, *** *p* < 0.001, ns *p* > 0.10. ^1^ Diet × period interaction. ^2^ Effect of covariate. ^3^ It refers to mean of diet × period interaction without inclusion of the preliminary period. ^4^ It is the standard error of the general mean. ^5^ Calculated according to Pulina et al. [14]. ^6^ Calculated according to Tedeschi et al. [15].

**Table 4:** Evolution of milk yield, fat-corrected milk yield, net energy for lactation, fat, protein, lactose, somatic cell count and urea in mid-lactating Sarda ewes fed high-starch and low-starch diets.

		Last Day of the Preliminary Period	Experimental Period (from 92 to 139 ± 11 DIM)								Effect			
	Diet		1	2	3	4	5	6	Mean ^3^	SEM ^4^	Diet	Period	D × P ^1^	π ^2^
Milk yield (kg/d)	HS	1.76	1.45	1.41	1.38	1.32	1.35	1.37	1.38	0.04	ns	ns	ns	***
	LS	1.78	1.54	1.48	1.41	1.42	1.41	1.40	1.44					
FCM (6.5%) (kg/d) ^5^	HS	1.72	1.40	1.39	1.38	1.30	1.33	1.38	1.36	0.04	**	ns	ns	***
	LS	1.70	1.56	1.49	1.45	1.42	1.47	1.46	1.47					
NE_L_ (Mcal NE/d) ^6^	HS	1.70	1.45	1.45	1.43	1.34	1.38	1.42	1.41	0.04	**	ns	ns	***
	LS	1.68	1.60	1.54	1.51	1.47	1.53	1.51	1.53					
Fat (%)	HS	5.8	6.2	6.3	6.5	6.5	6.4	6.6	6.4	0.07	**	*	ns	***
	LS	5.7	6.6	6.6	6.8	6.4	6.8	6.9	6.7					
Fat (g/d)	HS	102.0	89.3	89.0	89.1	84.2	86.1	89.6	87.9	2.6	**	ns	ns	***
	LS	99.3	101.7	96.8	95.8	91.4	97.5	96.4	96.6					
Protein (%)	HS	5.0	5.0	5.2	5.1	5.0	5.0	5.0	5.1	0.04	*	*	0.05	***
	LS	4.9	4.9	5.1	5.3	5.2	5.2	5.2	5.2					
Protein (g/d)	HS	86.5	71.9	72.7	70.0	65.4	67.2	68.2	69.2	2.0	*	ns	ns	***
	LS	86.5	75.1	75.0	74.0	73.8	74.1	73.8	74.3					
Lactose (%)	HS	4.6	4.5	4.5	4.5	4.4	4.5	4.5	4.5	0.04	ns	ns	ns	***
	LS	4.7	4.6	4.5	4.5	4.5	4.5	4.5	4.5					
SCC (log)	HS	3.1	3.1	3.0	3.1	2.8	2.8	2.9	3.0	0.06	ns	ns	ns	***
	LS	3.0	2.9	2.9	2.9	2.8	3.0	2.9	2.9					
Urea (mg/dL)	HS	38.9	45.5 ^a^	38.3	39.3	36.2	36.7	33.9	38.3	0.5	0.08	***	***	***
	LS	39.2	36.8 ^b^	39.2	38.1	36.9	38.5	35.1	37.4					

HS = High-starch diet; LS = Low-starch diet; DIM = Days in milk; FCM = Fat-corrected milk yield; NE_L_ = Daily milk net energy; NE = Net energy; SCC = Somatic cell count. ^a^,^b^ means within a column with a different superscript are considered significantly different (*p* < 0.001). * indicates *p* < 0.05, ** *p* < 0.01, *** *p* < 0.001, ns *p* > 0.10. ^1^ Diet × period interaction. ^2^ Effect of covariate. ^3^ It refers to mean of diet × period interaction without inclusion of the preliminary period. ^4^ It is the standard error of the general mean. ^5^ Calculated according to Pulina et al. [13]. ^6^ Calculated according to Tedeschi et al. [15].

**Table 5:** Metabolic and hormonal profile (mean ± SEM) in plasma of mid-lactating Saanen goats and Sarda ewes fed high-starch and low-starch diets.

		Goats		Ewes			Effect					
		HS	LS	HS	LS	SEM ^4^	Species	Diet	Period	S × D ^1^	S × P ^2^	D × P ^3^
Glucose (mg/dL)	Species × Diet	48.9	47.7	56.0	56.1	1.8	***	ns	ns	ns	ns	ns
	Species	48.3		56.0								
NEFA (mmol/L)	Species × Diet	0.2	0.2	0.2	0.1	0.02	*	ns	ns	*	ns	ns
	Species	0.2		0.1								
UREA (mg/dL)	Species × Diet	45.9	43.5	45.8	45.4	2.2	ns	ns	ns	ns	ns	ns
	Species	44.7		45.6								
GH (ng/mL)	Species × Diet	2.2	3.0	1.2	1.5	0.6	*	ns	*	ns	*	ns
	Species	2.6		1.4								
Insulin (µg/L)	Species × Diet	0.2	0.1	0.3	0.4	0.05	***	ns	0.08	ns	ns	ns
	Species	0.1		0.4								
IGF-I (ng/mL)	Species × Diet	101.0	100.7	130.0	109.4	13.6	ns	ns	ns	ns	ns	ns
	Species	100.8		119.7								
Leptin (ng/mL)	Species × Diet	25.0	24.4	11.3	12.7	2.2	***	ns	0.10	ns	0.06	ns
	Species	24.7		12.0								

HS = High-starch diet; LS = Low-starch diet; NEFA = Non-esterified fatty acids; GH = Growth hormone; IGF-I = Insulin-like growth factor I. * indicates *p* < 0.05, *** *p* < 0.001, ns *p* > 0.10. ^1^ Effect of Species × diet interaction. ^2^ Effect of species × period interaction. ^3^ Effect of diet × period interaction. ^4^ It is the standard error of the general mean.

## Data Availability

The data presented in this study are available on request from the corresponding author.
